# Tunable growth of one-dimensional graphitic materials: graphene nanoribbons, carbon nanotubes, and nanoribbon/nanotube junctions

**DOI:** 10.1038/s41598-023-31573-0

**Published:** 2023-03-15

**Authors:** Shuo Lou, Bosai Lyu, Jiajun Chen, Lu Qiu, Saiqun Ma, Peiyue Shen, Zhichun Zhang, Yufeng Xie, Qi Liang, Kenji Watanabe, Takashi Taniguchi, Feng Ding, Zhiwen Shi

**Affiliations:** 1 Key Laboratory of Artificial Structures and Quantum Control (Ministry of Education), Shenyang National Laboratory for Materials Science, School of Physics and Astronomy, Shanghai Jiao Tong University, Shanghai, 200240 China; 2 Collaborative Innovation Center of Advanced Microstructures, Nanjing University, Nanjing, 210093 China; 3 Centre for Multidimensional Carbon Materials, Institute for Basic Science, Ulsan, 44919 South Korea; 4 School of Materials Science and Engineering, Ulsan National Institute of Science and Technology, Ulsan, 44919 South Korea; 5 Research Center for Functional Materials, National Institute for Materials Science, 1-1 Namiki, Tsukuba, 305-0044 Japan; 6 International Center for Materials Nanoarchitectonics, National Institute for Materials Science, 1-1 Namiki, Tsukuba, 305-0044 Japan; 7 Tsung-Dao Lee Institute, Shanghai Jiao Tong University, Shanghai, 200240 China

**Keywords:** Nanoscale materials, Carbon nanotubes and fullerenes

## Abstract

Graphene nanoribbons (GNRs) and carbon nanotubes (CNTs), two representative one-dimensional (1D) graphitic materials, have attracted tremendous research interests due to their promising applications for future high-performance nanoelectronics. Although various methods have been developed for fabrication of GNRs or CNTs, a unified method allowing controllable synthesis of both of them, as well as their heterojunctions, which could largely benefit their nano-electronic applications, is still lacking. Here, we report on a generic growth of 1D carbon using nanoparticles catalyzed chemical vapor deposition (CVD) on atomically flat hexagonal boron nitride (h-BN) substrates. Relative ratio of the yielded GNRs and CNTs is able to be arbitrarily tuned by varying the growth temperature or feeding gas pressures. The tunability of the generic growth is quantitatively explained by a competing nucleation theory: nucleation into either GNRs or CNTs by the catalysts is determined by the free energy of their formation, which is controlled by the growth conditions. Under the guidance of the theory, we further realized growth of GNR/CNT intramolecular junctions through changing H_2_ partial pressure during a single growth process. Our study provides not only a universal and controllable method for growing 1D carbon nanostructures, but also a deep understanding of their growth mechanism, which would largely benefit future carbon-based electronics and optoelectronics.

## Introduction

Graphitic materials, i.e., sp^2^-hybridized carbon, includes 3D graphite, 2D graphene, 1D carbon nanotubes (CNTs) and graphene nanoribbons (GNRs), as well as 0D buckyballs. They own excellent electronic^1–3^, mechanical^4,5^ and thermal^6^ properties, and therefore hold great promise for a wide range of applications. Of particular interests, 1D CNTs and GNRs, due to their structurally-tunable bandgaps and ultra-high carrier mobility, show promise for future high-performance nanoelectronics^7,8^. Specifically, CNTs being either metallic or semiconducting determined by their chiralities, can serve as building blocks for miniaturized conducting wires and field-effect transistors (FET)^2,9^; GNRs with bandgaps inversely proportional to their width^10^ and spin-polarized zigzag-edge states^11–14^, are excellent candidate materials for spintronic devices.

Synthesis of those 1D graphitic materials forms the basis of their electronic applications. On the one hand, the synthesis of CNTs is rather mature as many growth methods have been developed since its discovery^15^ in 1991, such as catalytic chemical vapor deposition (CVD)^16^, laser ablation method^17^, and arc discharge method^18^. The most successful and widely used CNT growth method is the transition-metal-nanoparticle-catalyzed CVD, with which high-quality CNTs of uniform diameter and meters-long length have been achieved^16,19–22^. On the other hand, synthesis of micrometer-long, narrow, and smooth-edged GNRs turns to be rather difficult, although great efforts have been devoted^14,23–30^. A recent study shows that the nanoparticle-catalytic CVD can also be used to grow GNRs of high purity and high quality^31^. This establishes an important connection between the growth of CNTs and the growth of GNRs, and opens up opportunities to develop a generic method for growing both GNRs and CNTs with tunable yield, which is critical for future carbon-based electronic devices and circuits.

Here, we report a unified and controllable growth of GNRs and CNTs on h-BN substrate using nanoparticle-catalyzed CVD method, in which the ratio of GNRs/CNTs can be arbitrarily tuned. Experimentally, we observed a tunable and competing growth phenomenon between GNRs and CNTs: pure GNRs, pure CNTs, and mixed GNRs/CNTs can be selectively obtained through changing the growth temperature or hydrogen partial pressures. Theoretically, through calculating the free energy of formation of CNTs and GNRs, we reveal a competition between GNRs and CNTs during the nucleation stage: lower temperatures and higher hydrogen pressures energetically favor the growth of GNRs, whereas higher temperatures and lower hydrogen pressures support the growth of CNTs. The theory agrees well with the experimental results. Furthermore, under the guidance of the competing nucleation theory, we realized switching of GNR growth into CNT growth for an individual catalytic-nanoparticle. As a result, GNR/CNT intermolecular junctions are successfully fabricated through changing H_2_ pressures during a single growth progress.

## Results

### Growth and identification of GNRs and CNTs

**Figure 1:**
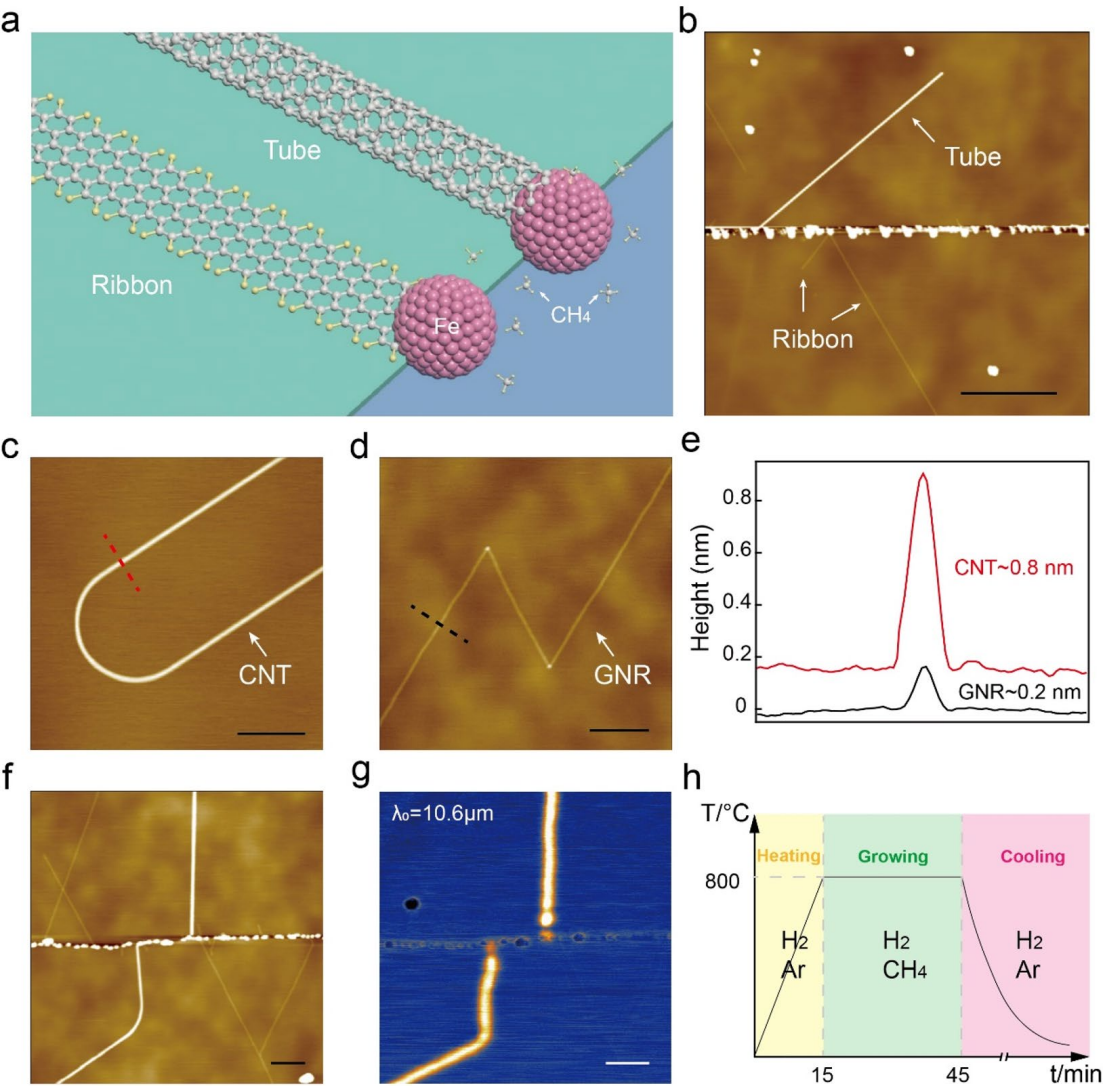
Growth and characterizations of GNRs and CNTs. (**a**) Schematic of GNR and CNT growth on h-BN substrate. The growth starts at Fe-nanoparticles, where methane molecules can decompose into atomic carbon, serving as source for the GNRs and CNTs growth. (**b**) AFM topography image of an as-grown sample with both GNRs and CNTs on h-BN. The higher (brighter) line corresponds to CNTs, and the lower (blurred) ones correspond to GNRs. Bright dots are Fe-nanoparticles. (**c**,**d**) AFM topography images of turns in CNTs and GNRs. A CNT tends to produce gentle-curved bends (**c**), while a GNR tends to produce sharp corners (**d**). This is due to the difference in their stiffness. (**e**) Height profiles of a CNT (red) and a GNR (black), taken along the dash lines in panel (**c**) and (**d**). The diameter of a nanotube is apparently larger than the thickness of a nanoribbon. (**f**,**g**) AFM topography image of CNTs and GNRs and the corresponding near-field infrared image of the same area. CNTs exhibit stronger response (brighter) than GNRs in the near-field optical image due to CNTs’ stronger optical conductivity. (**h**) Illustration of a typical growth process. Scale bars: 500 nm in (**b**); 100 nm in (**c**,**d**,**f**,**g**).

Figure 1a schematically illustrates the catalytic growth of GNRs and CNTs on an h-BN substrate. In prior to the growth, Fe nanoparticles are deposited onto h-BN substrates as catalysts. A typical growth process is depicted in Fig. 1h. Firstly, samples are heated to the growth temperature (~ 800 °C) in a CVD furnace with a flow of mixed Ar and H_2_. Then, CH_4_ gas is fed in as carbon source for the growth of CNTs or GNRs. A typical growth duration is 30 min. Lastly, the samples are cooled down to room temperature with protective gas of Ar and H_2_. The pressure during the whole process is atmospheric pressure (P_0_). (See methods for more growth details. Supplementary Information (Fig. S4) gives a schematic of the growth process of a CNT and a GNR).

Figure 1b shows an atomic force microscopy (AFM) topography image of the as-grown samples. Straight micrometer-long 1D structures grew out from catalytic Fe nanoparticles arranged along a h-BN step edge. We initially distinguish CNTs and GNRs through their heights (Fig. 1e) as flat GNRs (0.2–0.3 nm) are normally lower than tubular CNTs (~ 1 nm). Noted that the CNTs are single-walled. Another notable difference between tubes and ribbons is the shape of their turns. In general, GNRs (Fig. 1d) tend to produce sharper corners while CNTs tend to produce gentle-curved bends (Fig. 1c), as tubular CNTs have much larger bending stiffness than GNRs^25^. We can further identify the CNTs and GNRs using a scanning near-field optical microscopy (SNOM), where CNTs are much brighter than GNRs (Fig. 1g) due to CNTs’ larger optical conductivity than GNRs^32^. Figure 1f is the AFM topography image corresponding to the near-field infrared image. So far, we have successfully synthesized both GNRs and CNTs on h-BN substrates through a generic nanoparticle-catalyzed CVD method, and identified CNTs and GNRs through the differences in their height, shape and infrared response.

### Tunable population ratio between CNTs and GNRs

**Figure 2:**
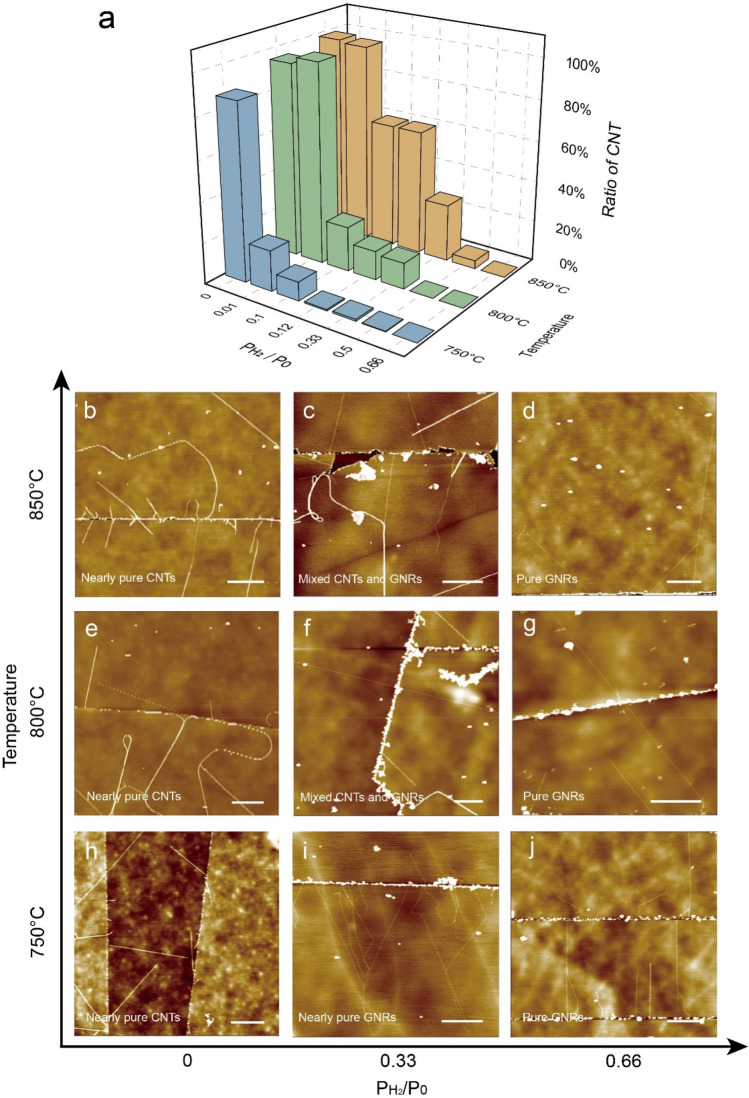
Variable ratio of GNRs and CNTs under different growth conditions. (**a**) Statistics of the proportion of CNTs as a function of growth temperatures and H_2_ pressures. (**b**–**j**) AFM images of representative samples grown under different conditions. High temperatures (850 °C) and low H_2_ pressures (P_H2_/P_0_ = 0) support the CNTs growth. Low temperature (750 °C) and high H_2_ pressures (P_H2_/P_0_ = 0.66) favor the GNRs growth. Scale bars: 500 nm.

It is rather surprising that the nanoparticle-catalyzed CVD growth can produce both planar GNRs and cylindrical CNTs simultaneously. To explore the tunability of the growth products, we carried out a series of growths with systematically varied temperatures and H_2_ pressures. The ratio of yielded CNT is plotted in Fig. 2a as 3D column diagram. From the diagram, we can see that high ratio of CNT appears at low H_2_ pressures and high growth temperature, whereas low ratio of CNT appears at high H_2_ pressures and low temperature. This implies that low H_2_ pressures and high temperature favor the growth of CNTs, and high H_2_ pressures and low temperature favor the growth of GNRs. To be more intuitive, we display in Fig. 2b–j AFM images of growth products at a few representative conditions. At low H_2_ pressures (P_H2_ = 0), the samples are nearly pure CNTs (Fig. 2b,e,h). With H_2_ pressures increasing to 0.33 P_0_, there are mixtures of GNRs and CNTs (Fig. 2c,f,i). When H_2_ pressures are high enough, only GNRs are obtained (Fig. 2d,g,j). At a moderate H_2_ pressures of 0.33 P_0_, one can clearly see more CNTs in high temperature samples, such as T = 850 °C (Fig. 2c), than low temperature samples (Fig. 2i). Thus, we demonstrate the large tunability of the growth products (pure GNRs, pure CNTs and mixtures samples) through modulating growth temperature and H_2_ pressures: lower H_2_ pressures and higher temperature tend to produce CNTs, and higher H_2_ pressure and lower temperature tend to produce GNRs.

### Competing nucleation theory of the catalytic-CVD growth

**Figure 3:**
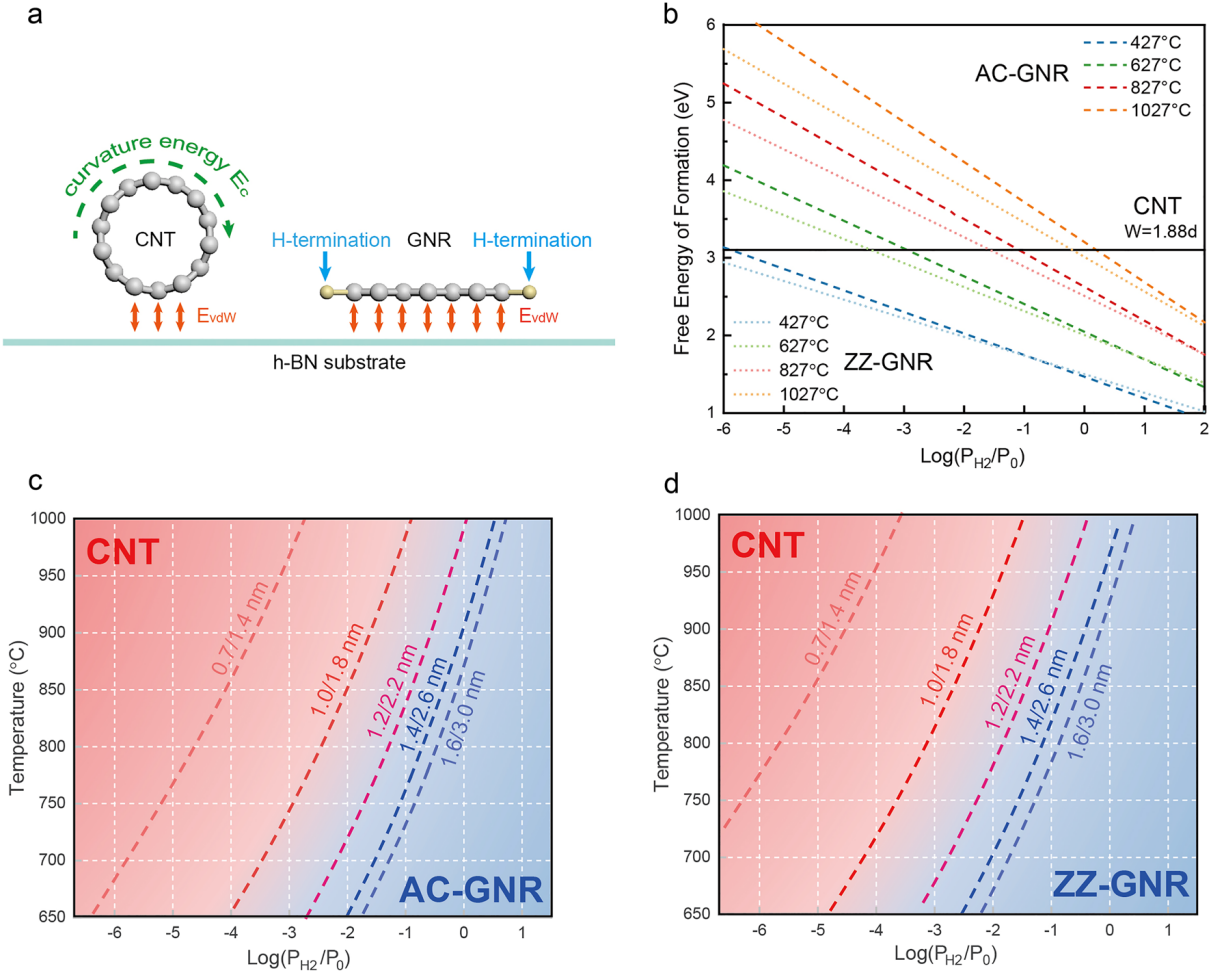
Competing nucleation between GNRs and CNTs. (**a**) Schematic of a tubular CNT and a planer GNR on h-BN substrate. (**b**) Free energy of formation of CNTs (black line) and zigzag/armchair (dot lines/dash lines) GNRs as a function of H_2_ pressures at a few representative temperatures. (**c**,**d**) Theoretical growth phase diagram. Red and blue regions stand for energetically favorable for growing CNTs and GNRs, respectively. The dashed lines represent equal probability of yielding GNRs and CNTs of different width and diameter. The diameter and the width of the compared CNTs and GNRs are denoted along the dash lines (CNTs’ diameter/ GNRs’ width).

In the growth, formation of either planar GNRs or tubular CNTs is largely determined in the nucleation stage. Therefore, an investigation of the microscopic process of the nucleation is required. During nucleation of either GNRs or CNTs, graphitic nano-islands will firstly form on the surface of catalyst nanoparticles. To create a CNT, the graphitic island will have to lift off from the particle to form a graphitic cap (or CNT nucleus) by introducing an extra curvature energy into the system^33,34^. Besides, the tubular structure of CNTs further leads to the reduction of their contact area with substrate (only the bottom part of CNTs as shown in Fig. 3a), and therefore loss of part of the van der Waals adhesion. As elaborated recently^34^, the driving force of tube formation is the reduction of the contact energy between the cap edge and the catalyst surface, which requires a strong interaction between the dangling atoms of the cap edge and the catalyst surface or the edge atoms of the cap cannot be passivated by any functional groups (such as –H, –OH or –O). Here, the presence of h-BN substrate opens up another channel to convert the gaseous carbon (CH_4_) into planar GNRs, which have a larger contact area with the h-BN substrate and thus a significant gain in adhesion energy compared to that in CNTs (Fig. 3a). Supplementary Information (Fig. S3) shows growth results on different substrates. But a pristine GNR has dangling bonds on both edges, whose formation energies are in the order of ~ 10 eV/nm^35^. These highly unstable edges must be passivated by tuning the precursor gases, and therefore the formation energies of the two edges of GNRs will depends on the H_2_ pressure under our CVD condition. In general, CNT formation would be favorable if the partial pressure of H_2_ gas is small and GNRs might be formed with large H_2_ pressure.

Theoretically, nucleation of either CNTs or GNRs depends largely on their free energy of formation. In the following, through comparison of their formation energies, a theoretical growth diagram of GNR vs. CNT was achieved and a competing nucleation mechanism was revealed. We first calculated the free energies of formation of both GNRs and CNTs at different temperatures and under different pressures of H_2_, by including the adhesion energy with substrate, curvature energy and H-passivated edge energy (Fig. 3b). In the experiments, the average width (W) of the synthesized AC-GNR is ~ 2.2 nm, the average diameter (d) of the CNT is ~ 1.2 nm, and therefore, the width to diameter ratio is ~ 1.88. We did the DFT calculations of GNRs and CNTs with such effective ratio W = 1.88 d. The free energy of CNTs remains a constant, whereas that of GNRs changes systematically with growth temperature T and hydrogen pressure P_H2_, due to the corresponding change of edge formation energy in GNRs. Note that at certain condition where the energy lines of GNRs and CNTs cross, the two species have the same free energy of formation. This provides possibilities to realize switching between formations of GNRs and CNTs in the growth. Based on the calculation of the free energy of formation, we draw a nucleation phase diagram of GNRs vs. CNTs (Fig. 3c,d). From the phase diagram, one can see that the nucleation of either GNRs (blue region) or CNTs (red region) is determined by the temperature and H_2_ pressures in the reactor and the width/diameter of the GNR/CNT. The lower temperature and the higher partial pressures of H_2_, the more stable the GNRs. Meanwhile, in the condition of higher temperature and lower H_2_ partial pressures, the nucleation of CNTs is more energetically favorable (more calculation details see methods). The theoretical phase diagram of competing nucleation agrees well with the experimental growth results.

### GNR/CNT intramolecular junctions

**Figure 4:**
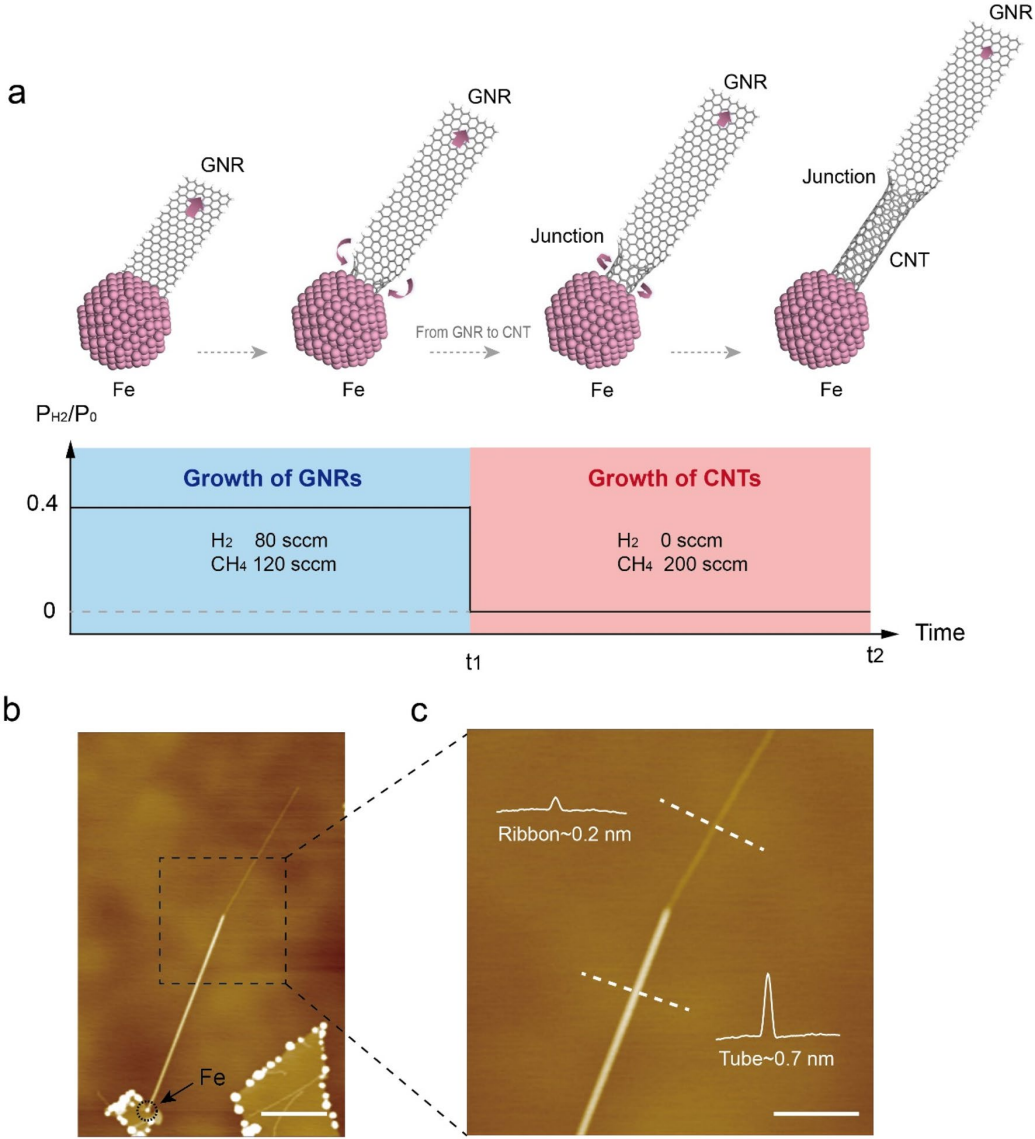
Growth of GNR/CNT junctions. (**a**) Schematic of growth of GNR/CNT junctions through abrupt change of H_2_ pressure. The product switches from ribbon to tube, which yields a junction structure in the middle. (**b**) Topography of a typical GNR/CNT junction structure. (**c**) A zoom-in AFM image of the joint region in the dashed box in panel (**b**). Height profiles taken along the dashed lines show the tube height ~ 0.7 nm and ribbon height ~ 0.2 nm. Scale bars: 200 nm in (**b**); 100 nm in (**c**).

Under the inspiration of the competing nucleation theory, we successfully grow a novel GNR/CNT heterojunction structure. Based on the theory, varying H_2_ pressures or temperature can tune the growth from GNR favorable to CNT favorable. Thus, heterojunction structures consisting of GNR section and CNT section can in principle be synthesized through adjusting growth conditions during a single growth process. Here, by abruptly changing the H_2_ pressures in a single growth process, we successfully synthesized GNR/CNT heterojunctions. Figure 4a schematically shows the steps for growing a GNR/CNT junction. First, mixed gases of CH_4_ (120 sccm) and H_2_ (80 sccm) were injected to grow the GNR section in the first stage. Then, all remaining gases in the CVD furnace were rapidly pumped out. After that, only CH_4_ gas was supplied to grow CNT section in the second stage. Figure 4b displays an AFM image of an as-grown CNT/GNR junction sample, and Fig. 4c is a zoom-in image of the area denoted by the square box in Fig. 4b. It clearly shows two distinct sections with different heights: one with height of ~ 0.7 nm and the other one with height of ~ 0.2 nm, revealing that this sample consists of both CNT and GNR sections. More GNR/CNT junctions structures can be found in Supplementary Information (Fig. S1). The success in fabrication of GNR/CNT junctions further demonstrates the controllability of our nano-particle catalyzed CVD growth method and also verifies the competing nucleation theory. In addition, the obtained GNR/CNT heterojunction can serve as building blocks for future carbon-based electronic and optoelectronic devices, such as field-effect transistors, logic gates and high-performance photodetectors^36^. We would like to note that the transition from GNR to CNT allows the CNT growth without a cap formation. Typically, the chirality of the CNT depend on the atomic structure of its cap, which generally leads to a random distribution of chiralities^37^. Our previous study clearly showed that the controllable synthesis of GNR is possible, thus this study provides a feasible way to grow chirality controlled CNTs.

## Discussion

We report the growth of 1D graphitic nanomaterials on h-BN substrates using a unified catalytic-CVD method, with which pure CNTs, pure GNRs, mixed CNTs/GNRs, and CNT/GNR heterojunctions are successfully achieved. Experimentally, we found that the population ratio of CNT or GNR can be efficiently tuned through modulating growth temperature or H_2_ pressure. Theoretically, we calculated the free energy of formation, and revealed a competition between GNRs and CNTs during the nucleation stage, which agrees well with our experimental results. Under the guidance of the competing nucleation theory, a novel GNR/CNT junction structure was successfully synthesized through abrupt change of H_2_ pressures within a single growth process. The reported tunable growth of GNRs and CNTs through a simple catalytic-CVD method provide not only a deep understanding of the growth mechanism of 1D graphitic materials, but more importantly an exciting approach for synthesizing building blocks for future carbon-based nanoelectronics.

## Methods

### CVD growth of GNRs, CNTs and GNR/CNT intramolecular junctions

Hexagonal-BN flakes were mechanically exfoliated onto SiO_2_/Si as growth substrates. Next, h-BN on SiO_2_/Si substrates were annealed at 300 °C with hydrogen to remove organic contaminations. Then, 0.5–1 Å thick Fe film, as catalyst, was deposited onto the substrates using thermal evaporation at a rate of ~ 0.05 Å/s. The substrates were loaded into a furnace tube (Anhui BEQ Equipment Technology), followed by pumping out the remaining air in the tube. Then, the substrates were gradually heated up to the growth temperature (typically, 750/800/850 °C) for 15 min under an Ar/H_2_ flow (200 sccm). After reaching the target growth temperature, a CH_4_/H_2_ flow (200 sccm) was injected into the chamber for the growth of GNRs and CNTs, and the growth temperature was kept for 30 min. The ratio of hydrogen was tuned to obtain different ratios of yielded GNRs and CNTs. For GNR/CNT intramolecular junctions growth, a single growth process was consist of two stages. In the first stage, a CH_4_/H_2_ flow with high partial pressure of hydrogen was injected to grow the GNR section. In the second stage, pure CH_4_ gas was supplied to grow the CNT section. Before starting the second stage, all remaining gases in the CVD furnace of the first stage were rapidly pumped out. Finally, the systems were cooled down to room temperature under a protective hydrogen and argon atmosphere (Supplementary Information).

### Atomic force microscopy (AFM)

A commercial AFM (Cypher S, Asylum Research, Oxford Instruments) was used to image the topography of the as-grown samples. All samples were scanned in AC mode (tapping mode) in ambient conditions. AFM probes of RTESPA-150 and PFQNE-AL were typically used for the scanning.

### Scanning near-field optical microscopy (SNOM)

A home-built SNOM setup (main components include: Innova AFM, Bruker; L3S CO_2_ laser, Access Laser and KLD-0.1-J1 MCT detector, Kolmar) was used to detect the infrared response of the as-grown samples. Infrared beam of 10.6 μm was focused onto the apex of a gold-coated AFM tip (MikroMasch HQ:NSC35/Cr-Au). The near-field at the apex was strongly enhanced by the tapped tip, leading to a strong local interaction of light with material that underneath the tip. The scattered light with local optical information of the sample was collected by an infrared detector. Near-field optical images with spatial resolution better than 20 nm can be achieved with sharp AFM tips.

### Calculations of free energy of formation and growth phase diagram

In this work, we mainly concentrated on the role of the presence of h-BN substrate and H_2_ partial pressure during the growth, which determines either GNR or CNT is the thermodynamically favorable species to be nucleated. To include the influence of h-BN substrate and H_2_ partial pressure on the selective nucleation of GNR or CNT, we first considered their difference in the van der Waals adhesions with h-BN substrate, as shown in Fig. 3a where a GNR has a large contact area with the substrate but only the bottom part of a CNT is in contact with the substrate. For CNT, there is extra curvature energy; while for GNR, there is extra hydrogen termination (Fig. 3a). Evaluating all the three energy terms, we obtained the phase diagram that shows, under the growth conditions (temperature and H_2_ pressure), which one (GNR or CNT) is energetically more favorable, and is more likely to be synthesized.

All DFT calculations were performed using the Vienna Ab initio Simulation Package (VASP)^38–40^ with projected augmented wave (PAW) method^41^. The generalized gradient exchange–correlation functional approximation (GGA)^42^ was employed with the D3 dispersion correction^43^ in order to precisely describe van der Waals interactions. The plane-wave cutoff energy was set to be 600 eV and the Brillouin zone was sampled using Monkhorst–Pack k-mesh with a separation criterion of 0.02^44^. Criteria for energy and force convergence were set to be 10^–4^ eV and 10^–2^ eV/Å, respectively.

To compare the thermodynamic stability of GNRs and CNTs at growth condition, we included the influence of temperature and pressure to the system, and estimated the free energy of GNR formation by the following equation^45,46^:

1 $$\Delta {G}_{f}({\text{GNR}})={E}_{f}({\text{GNR}})+{\Delta F}_{vib}-\frac{1}{2}{N}_{H}\times {\mu }_{{H}_{2}},$$

where ${E}_{f}({\text{GNR}})$, ${\Delta F}_{vib}$ ${N}_{{\text{H}}}$ and ${\mu }_{{H}_{2}}$ are the ground state (0 K) formation energy of GNRs, vibrational contribution of the hydrogen termination at GNR edge, number of hydrogen atoms at edge, and hydrogen chemical potential in gas phase. The vibrational contribution term was estimated with the following equation^47^:

2 $$\Delta {F}_{{\text{vib}}}={\sum }_{\omega }h\omega \left(\frac{1}{2}+\frac{1}{{e}^{\beta h\omega }-1}\right)-{k}_{B}T\left[\frac{\beta h\omega }{{e}^{\beta h\omega }-1}-{\text{ln}}(1-{e}^{-\beta h\omega })\right],$$

where $\omega$ and *h* are the phonon frequency and Plank’s constant, and $\beta ={({k}_{B}T)}^{-1}$. The third term on the right of Eq. (1) was estimated by bellow equation^46^:

3 $${\mu }_{{H}_{2}}={H}^{0}(T)-{H}^{0}(0{\text{ K}})-T{S}^{0}(T)+{k}_{B}Tln\frac{{P}_{{H}_{2}}}{{P}_{0}},$$

where, the standard values, ${H}^{0}(T)$, ${H}^{0}(0{\text{ K}})$, and ${S}^{0}(T)$ are obtained from chemical tables^48^, ${P}_{0}$ is atmospheric pressure, and ${P}_{{H}_{2}}$ is experimental hydrogen pressure.

## Supplementary Information

Supplementary Information.

## Data Availability

The datasets used during the current study available from the corresponding author on reasonable request.
