# Supplementary material for: Tunable growth of one-dimensional graphitic materials: graphene nanoribbons, carbon nanotubes, and nanoribbon/nanotube junctions
Source: Sci Rep. 2023 Mar 15;13:4328. doi: 10.1038/s41598-023-31573-0 (PMC10017793; doi:10.1038/s41598-023-31573-0)
Supplement: Supplementary file 1 — Supplementary Information. [file 41598_2023_31573_MOESM1_ESM.docx]

**Supplementary information**

**Tunable growth of one-dimensional graphitic materials: graphene nanoribbons, carbon nanotubes, and nanoribbon/nanotube junctions**

Shuo Lou^1,2†^, Bosai Lyu^1,2†^, Jiajun Chen^1,2†^, Lu Qiu^3,4†^, Saiqun Ma^1,2^, Peiyue Shen^1,2^, Zhichun Zhang^1,2^, Yufeng Xie^1,2^, Qi Liang^1,2^, Kenji Watanabe^5^, Takashi Taniguchi^6^, Feng Ding^3,4*^, Zhiwen Shi^1,2,7*^

^1^Key Laboratory of Artificial Structures and Quantum Control (Ministry of Education), Shenyang National Laboratory for Materials Science, School of Physics and Astronomy, Shanghai Jiao Tong University, Shanghai 200240, China.

^2^Collaborative Innovation Center of Advanced Microstructures, Nanjing University, Nanjing 210093, China.

^3^Centre for Multidimensional Carbon Materials, Institute for Basic Science, Ulsan 44919, South Korea.

^4^School of Materials Science and Engineering, Ulsan National Institute of Science and Technology, Ulsan 44919, South Korea

^5^Research Center for Functional Materials, National Institute for Materials Science, 1-1 Namiki, Tsukuba 305-0044, Japan.

^6^International Center for Materials Nanoarchitectonics, National Institute for Materials Science, 1-1 Namiki, Tsukuba 305-0044, Japan.

^7^Tsung-Dao Lee Institute, Shanghai Jiao Tong University, Shanghai, 200240, China.

^†^These authors contributed equally to this work.

*Correspondence to: f.ding@unist.ac.kr, zwshi@sjtu.edu.cn

**1. AFM images of more GNR/CNT junctions**

**2. The relation of CNTs diameter and catalyst particle size**

**3. Growth results on different substrates.**

**4. The growth process of a CNT and a GNR.**

**1. AFM images of more GNR/CNT junctions**


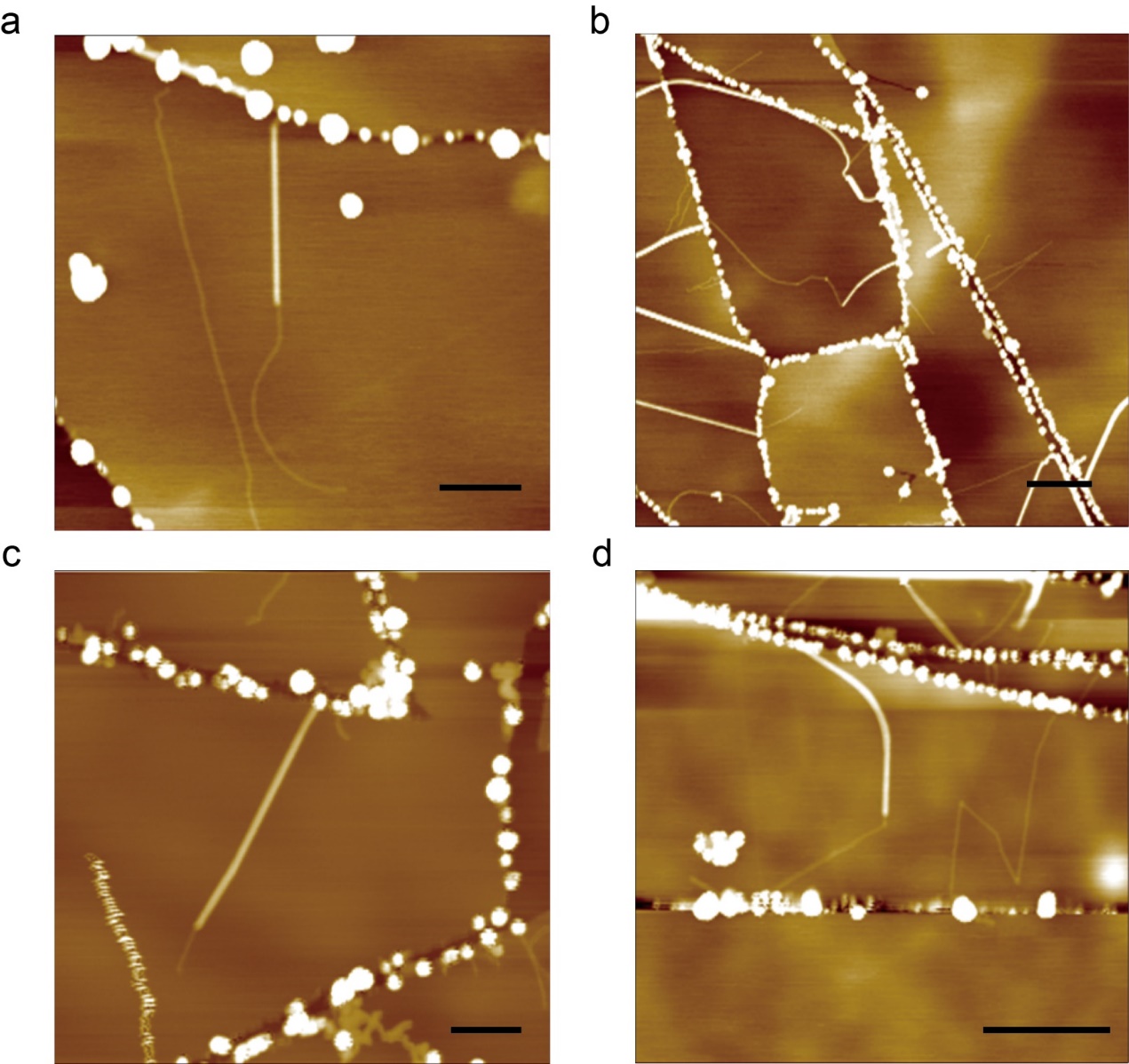


**Figure S1. More as-grown GNR/CNT junction structures.** Scale bars: 100nm in panel a, c; 200nm in panels b and d.

**2. The relation of CNTs diameter and catalyst particle size.**


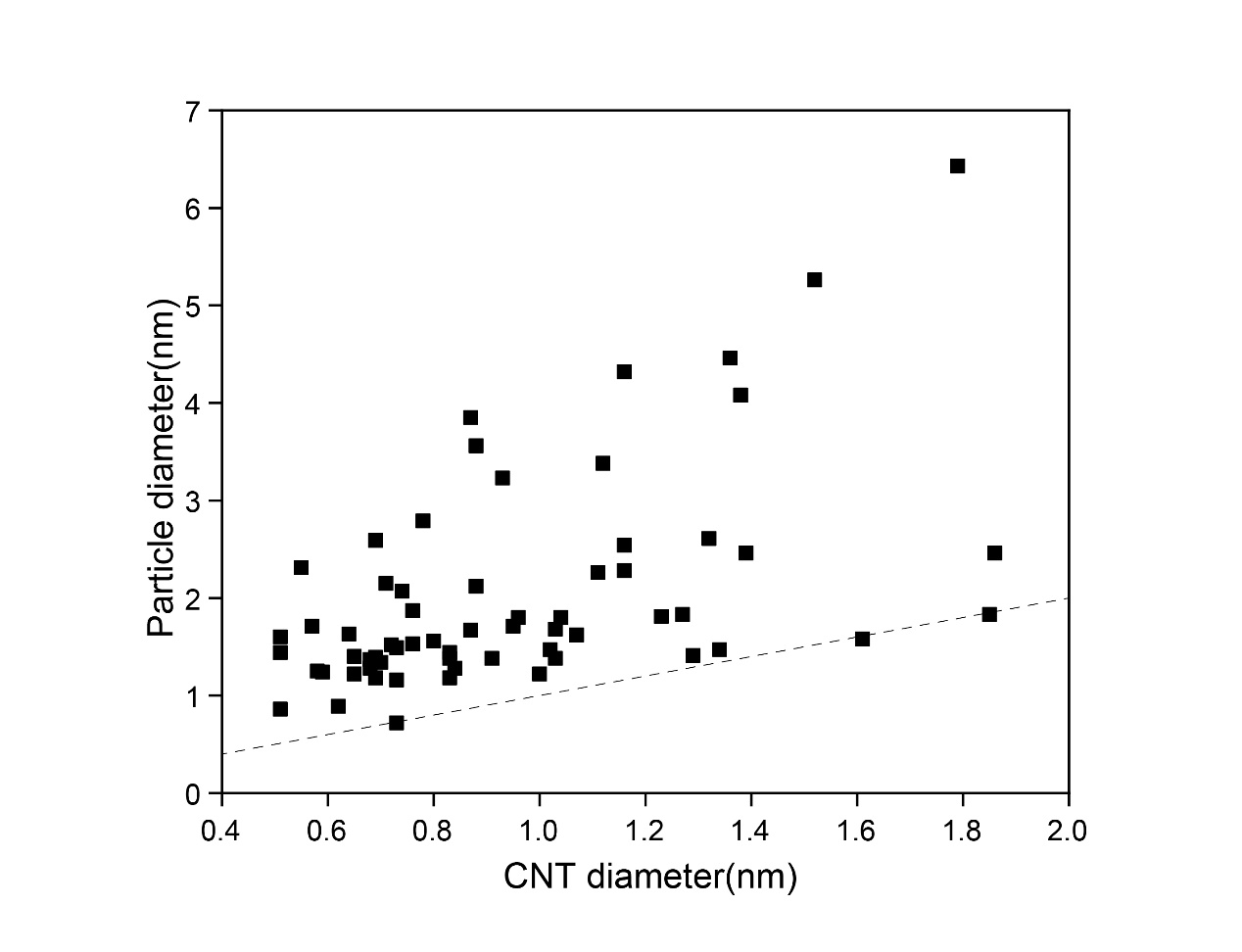


**Figure S2. The relation between CNTs diameter and catalyst particle size.** Dash line indicates that the CNT diameter is equal to the catalyst particle diameter. Most of catalyst particles are bigger than the CNT diameter.

**3. Growth results on different substrates.**

We have tried other substrates for the growth of GNRs and CNTs. As a substrate with atomically flat surface, mica can support the nucleation of GNRs, but GNRs grown on mica are typically very short and not straight (figure S3 (a)). This is because the friction between mica and the GNR is not low enough, as they have very different lattice structures.

We have also tried to grow GNRs on SiO_2_/Si substrate that is not atomically flat. Figure S3 (b) shows that only CNTs can be found without any GNRs on the SiO_2_/Si substrate. This is because the friction between the GNR and the substrate is too high that hinders the GNR growth, as rough surfaces typically induce very large friction force.


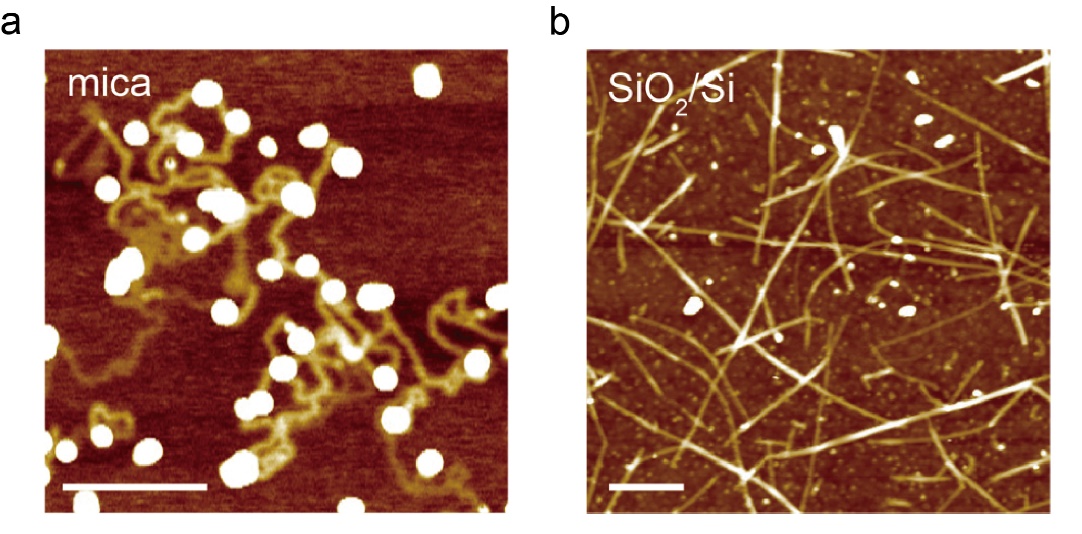


**Figure S3. Growth results on different substrates.** a, AFM image of as-grown GNRs on mica substrate. b, AFM image of CNTs on SiO_2_/Si substrate. Scale bar: 200 nm.

**4. The growth process of a CNT and a GNR.**


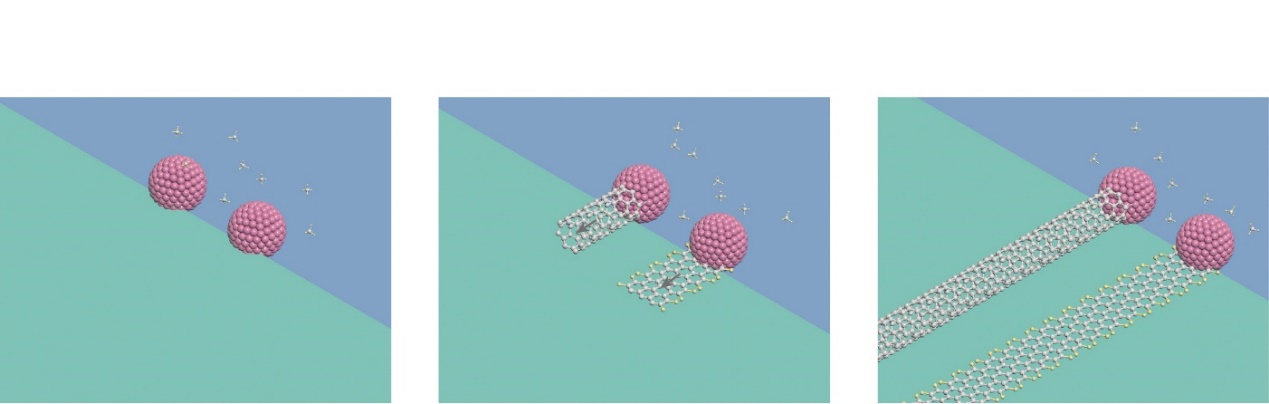


**Figure S4**. **Schematic of the growth process of a CNT and a GNR.** First, methane molecules are decomposed and dissolved into Fe nanoparticles trapped at an h-BN step edge. Then, when over-saturated, tube and/or ribbon structures are nucleated. Following a base-growth model, the grown GNR and/or CNT are push away and slide on the h-BN substrate. Finally, long CNT and/or GNR are achieved.
